# Association Between Problematic Internet Use, Autistic Traits, and Psychological Distress Among Adults: a Cross-Sectional Survey

**DOI:** 10.1192/j.eurpsy.2026.10500

**Published:** 2026-07-31

**Authors:** M. Floris, C. Gentili

**Affiliations:** 1 General Psychology, University of Padova; 2 Padova Neuroscience Center, Padova, Italy

## Abstract

**Introduction:**

Problematic Internet Use (PIU) is an emerging public health concern, and has been associated with various mental health conditions, including autistic traits. However, PIU remains poorly understood, and the role of psychological distress as contributing factor has not been fully clarified.

**Objectives:**

This study examined prevalence and associations of autistic traits, Internet and smartphone use, and substance use in adults, while accounting for psychological distress.

**Methods:**

A cross-sectional online survey was conducted among 419 Italian adults aged 18-65. Autistic traits (Autism Spectrum Quotient, AQ), Internet use (Questionario sull’Uso e Abuso di Internet-2, UADI-2), smartphone use (Mobile Phone Problematic Use Scale for Adults, MPPUS), substance use (Alcohol, Smoking and Substance Involvement Screening Test, ASSIST) were assessed, with psychological distress (Kessler Psychological Distress Scale, K10) as covariate. Analyses included descriptive statistics, correlations, and MANCOVA, with exploratory ANCOVAs adjusted for sociodemographic variables.

**Results:**

The sample is characterized by low-autistic traits (85%, n = 358), mild psychological distress (M = 22.32, SD = 8.23), normal Internet use (M = 56.97, SD = 15.64) and moderate smartphone use (M = 53.30, SD = 16.01). Younger participants (18–24 years) showed abusive Internet use and high use of smartphone. Correlational analyses showed that autistic traits were positively associated with Internet use (ρ = 0. 22 – 0.51, all *p* < .03) and smartphone use (ρ = 0. 24 – 0.42, all *p* < .01). Psychological distress positively correlated with Internet use (ρ = 0. 28 – 0.59, all *p* < .006) and smartphone use (ρ = 0. 27 – 0.58, all *p* < .009) across age groups, and with tobacco and alcohol use in younger participants. In MANCOVA, both autistic traits (V= 0.11, F(4, 413) = 13.44, *p* < .001) and psychological distress (V= 0.28, F(4, 413) = 41.88, *p* < .001) were associated with outcomes. ANCOVAs showed that autistic traits were significantly associated only with PIU (Internet: F(1, 361) = 61.78, *p* < .001; smartphone: F(1, 361) = 33.01, *p* < .001), whereas psychological distress had a significant effect on Internet use (F(1, 361) = 152.15, *p* < .001), smartphone use (F(1, 361) = 156.89, *p* < .001), and substances use (alcohol: F(1, 361) = 23.32, *p* < .001; tobacco: F(1, 361) = 39.31, *p* < .001).

**Conclusions:**

Autistic traits and psychological distress were significantly associated with PIU, with distress showing stronger associations. PIU appears specifically linked to autistic traits, whereas only psychological distress is linked to substance use, suggesting different pathways. Further efforts should prioritize the assessment of PIU in young adults and the development of coping-skills and parenting-focused interventions.

**Disclosure of Interest:**

None Declared

